# A digital pathology workflow for the segmentation and classification of gastric glands: Study of gastric atrophy and intestinal metaplasia cases

**DOI:** 10.1371/journal.pone.0275232

**Published:** 2022-12-30

**Authors:** Panagiotis Barmpoutis, William Waddingham, Jing Yuan, Christopher Ross, Hamzeh Kayhanian, Tania Stathaki, Daniel C. Alexander, Marnix Jansen

**Affiliations:** 1 Department of Computer Science, Centre for Medical Image Computing, University College London, London, United Kingdom; 2 Department of Pathology, UCL Cancer Institute, University College London, London, United Kingdom; 3 Department of Electrical and Electronic Engineering, Imperial College London, London, United Kingdom; Sant Longowal Institute of Engineering and Technology, INDIA

## Abstract

Gastric cancer is one of the most frequent causes of cancer-related deaths worldwide. Gastric atrophy (GA) and gastric intestinal metaplasia (IM) of the mucosa of the stomach have been found to increase the risk of gastric cancer and are considered precancerous lesions. Therefore, the early detection of GA and IM may have a valuable role in histopathological risk assessment. However, GA and IM are difficult to confirm endoscopically and, following the Sydney protocol, their diagnosis depends on the analysis of glandular morphology and on the identification of at least one well-defined goblet cell in a set of hematoxylin and eosin (H&E) -stained biopsy samples. To this end, the precise segmentation and classification of glands from the histological images plays an important role in the diagnostic confirmation of GA and IM. In this paper, we propose a digital pathology end-to-end workflow for gastric gland segmentation and classification for the analysis of gastric tissues. The proposed GAGL-VTNet, initially, extracts both global and local features combining multi-scale feature maps for the segmentation of glands and, subsequently, it adopts a vision transformer that exploits the visual dependences of the segmented glands towards their classification. For the analysis of gastric tissues, segmentation of mucosa is performed through an unsupervised model combining energy minimization and a U-Net model. Then, features of the segmented glands and mucosa are extracted and analyzed. To evaluate the efficiency of the proposed methodology we created the GAGL dataset consisting of 85 WSI, collected from 20 patients. The results demonstrate the existence of significant differences of the extracted features between normal, GA and IM cases. The proposed approach for gland and mucosa segmentation achieves an object dice score equal to 0.908 and 0.967 respectively, while for the classification of glands it achieves an F1 score equal to 0.94 showing great potential for the automated quantification and analysis of gastric biopsies.

## Introduction

Gastric cancer is a major public health issue. According to the latest global cancer statistics, it remains one of the most common cancers and it is one of the leading causes of cancer-related deaths mainly due to its often-late stage of diagnosis [1]. The risk factors of gastric cancer include Helicobacter pylori infection, salt intake, smoking, alcohol consumption, family history of gastric cancer, gastric atrophy (GA) and intestinal metaplasia (IM) [1–3]. In particular, several studies suggest that GA and IM of the mucosa of the stomach are major precursor lesions of gastric cancer [4, 5]. For this reason, early and effective diagnosis of GA and IM is a crucial step to prevent gastric cancer. The presence of GA is defined as the loss of glands in the gastric mucosa and IM is considered to be an advanced stage of atrophy [6]. In the latter, the metaplastic glands replace the native gastric glands and Paneth cells, goblet cells and absorptive cells appear. Widely used diagnostic methods for GA and IM include endoscopic and histological diagnosis. Endoscopic diagnosis of extensive GA and IM is effortless, but there are difficulties in making the diagnosis of mild GA and IM cases. Therefore, a biopsy confirmation for staging suspected cases of GA and IM remains the gold standard approach. To this end, the classification Sydney System was introduced in 1990. This was updated in 1996 introducing a visual analogue scale for evaluating the severity of histological staging [6]. Based on this protocol, the morphological features of GA and IM are identified and are visually inspected by histopathologists. Furthermore, for the prognosis of gastric cancer risk in cases with GA and IM the histological Operative Link for Gastritis Assessment (OLGA) and Operative Link on Gastric Intestinal Metaplasia (OLGIM) systems have been adopted [7]. These systems use biopsies from at least two sites (antrum and corpus) and the visual analogue scales recommended by the updated Sydney system, and correlate histopathological staging with cancer risk.

However, the visual qualitative assessment of glands by histopathologists is a labour-intensive and time-consuming task. Thus, the automated precise segmentation of glands from the histological images plays an important role in glandular morphology analysis, which is a crucial criterion for the effective detection and management of GA and IM. To date, no generally applicable end-to-end digital pathology approach has been proposed and applied for gastric gland segmentation, classification and study of gastric atrophy and intestinal metaplasia. Towards this end, in this paper, we propose a digital pathology framework which aims to extend our previous work for gastric gland segmentation and classification and analysis of gastric tissues based on Hematoxylin and Eosin (H&E) -stained Whole Slide Images (WSI). More specifically, this paper makes the following contributions:

- We propose the end-to-end GAGL-VTNet model consisting of two parts: The segmentation part, named GAGL-Net (GAstric GLands-Net) extracts both global and local features for gastric gland segmentation. The classification part, named IMGL-VTNet (Intestinal Metaplasia gastric GLands-Vision Transformer Net) adopts a multi-scale deformable transformer for the classification of glands into normal and IM glands.
- We introduce a weakly-supervised approach combining an energy minimization technique and a U-Net model for mucosa segmentation.
- We analyze the segmented glands and mucosa which demonstrate significant differences between the extracted features of normal, gastric atrophy and intestinal metaplastic cases. Through this analysis we translate the analogue visual scales described in the Sydney system into a reproducible set of mathematical values regarding the number and area that the detected glands cover.
- We have created the GAGL dataset consisting of 85 WSI of normal, gastric atrophy and intestinal metaplastic cases, collected from 20 patients.

## Related work

Recent years have witnessed a tremendous progress in medical image analysis. The most common application areas of digital pathology image analysis, include image synthesis and reconstruction, registration, segmentation, abnormality detection, disease grading and classification, as well as computer-aided diagnosis. Given the various challenges, several techniques and methods have been developed, based on either hand-crafted or deep learning features. Hand-crafted developed approaches are based on grayscale density, color, texture and shape information extracting low-level or mid-level set of features [8–10]. On the other hand, more sophisticated methods [11, 12] and deep-learning techniques including convolutional neural networks (CNNs) [13] and visual transformers (VTs) [14, 15] have been developed aiming to address medical image challenges by extracting high-level features directly from the data.

Medical image segmentation plays a vital role in image analysis and is important for computer-aided diagnosis and treatment planning. To this end, numerous methods have been proposed in literature for gland segmentation. Traditional methods include approaches that rely on decomposing the images into a set of primitive objects [16] for the identification and association of epithelial nuclei [17] and gland lumen [18]. A previous study [19] used prior knowledge of spatial connectivity and arrangement of neighboring epithelial nuclei. Each glandular structure was considered as a polygon of a random number of vertices which represents approximate locations of the epithelial nuclei. Similarly, for gland detection, Biomedical Imaging Laboratory [20, 21] developed a multi-step methodology based on the identification of epithelial cells and morphological operations.

The need for capturing representative features directly from data has led to the development of deep learning methods aiming to address the medical segmentation problems by extracting knowledge directly from data. Thus, various approaches have been developed for gland segmentation adopting various strategies and techniques. Among these methods, Chen et al. [22] proposed a deep contour-aware network aiming to focus on the boundaries’ segmentation among glands. This was achieved by a fully convolutional network with two different branches and three weighted auxiliary classifiers to enhance the discrimination capability and strengthen the training optimization process. Furthermore, Xu, et al. [23] combined foreground segmentation with edge detection and object detection using a deep multichannel side supervision model for instance segmentation in gland histology images. Other methods focus on the design of loss functions. Graham et al. [24] aimed at retaining maximal information, that is essential for segmentation by minimal information loss units, incorporating the original downsampled image into the residual unit. On the other hand, Yan, et al. [25] trained a unified model through a shape-preserving loss function for both pixel-wise gland segmentation and boundary detection. Similarly, Ding et al., [26] proposed a three-class classification model aiming to achieve boundary segmentation while retaining the global information. The majority of the prior gland segmentation research has applied to the dataset provided by the MICCAI 2015 Gland Segmentation Challenge Contest [21], focusing on colon histological images. Thus, only a limited number of studies have carried out experiments using prostate and breast histological images.

On the other hand, medical image classification aims to distinguish and assign labels to medical images according to diseases’ severity and to clinical pathologies. The development of hand-crafted machine learning methods requires the manual selection and extraction of features, a procedure that is time-consuming and varies depending on different objects. In contrast, deep neural network methods, which are inspired by actual neural networks in the brain and how they process patterns, aim to replace the manual feature acquisition and are used to design complex generalized systems. These models comprise various layers translating input images to give the desired outputs. However, although plenty of advanced approaches have been developed for histopathological image classification tasks [27], there is a limited research on the classification of gastric glands.

The remainder of this paper is organized as follows: In Section 2, we describe the workflow used in the experimental analysis, as well as the proposed methodology for gland and mucosa segmentation. The experimental results of our study are given in Section 3, while conclusions are drawn in Section 4.

## Materials and methods

**Fig 1:**
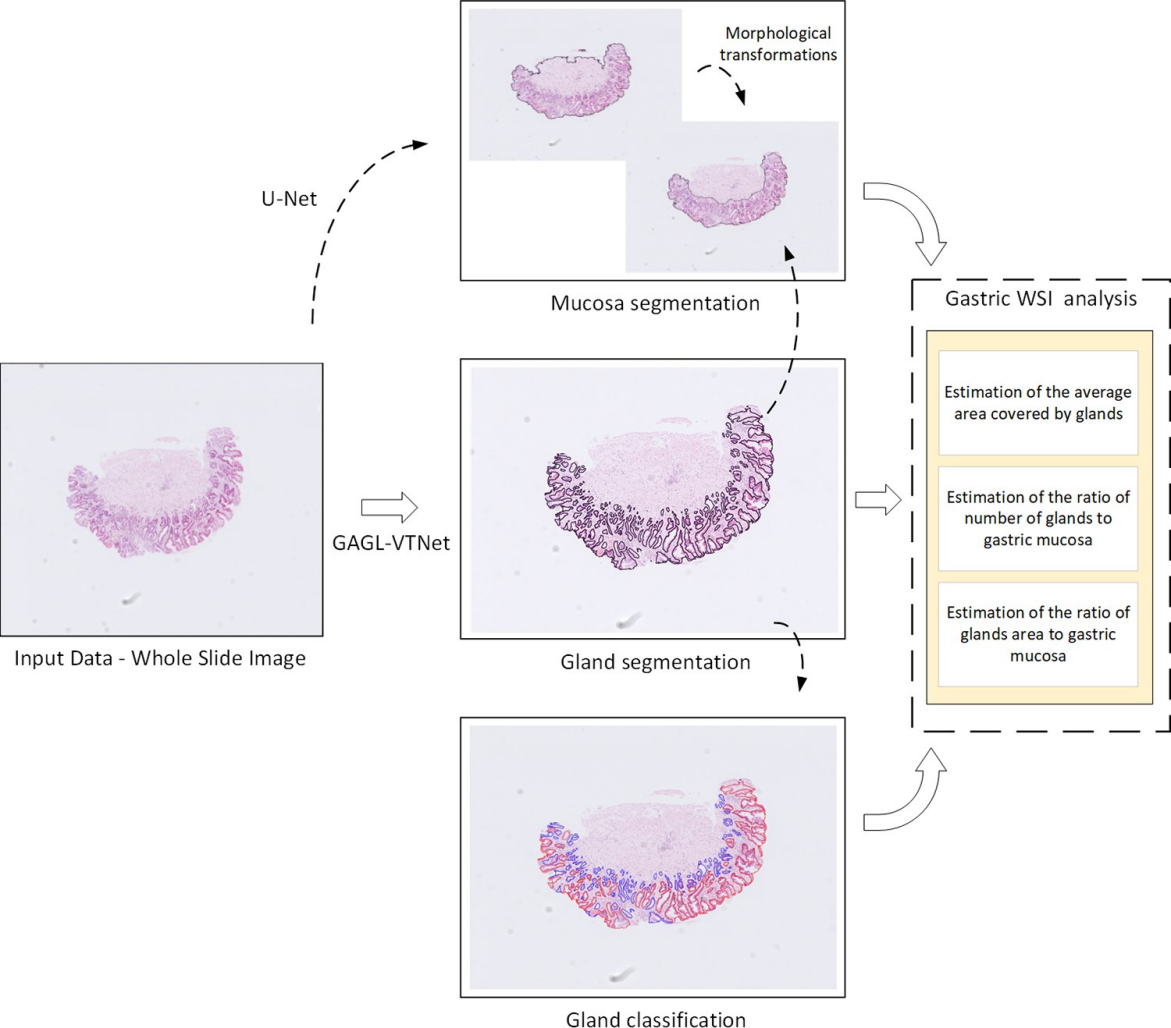
The proposed framework for gastric gland segmentation, classification and gastric WSI analysis.

The framework of the proposed methodology for the gastric gland and mucosa segmentation is shown in Fig 1. Initially, a WSI is fed into both the U-Net model for tissue segmentation and artifacts’ rejection and the GAGL-VTNet model for gland segmentation and gland classification. Then, based on the combination of tissue and gland segmented masks, identification of mucosa is performed. Finally, gastric gland features are extracted and gastric tissues are analyzed towards the identification of significant differences between normal, gastric atrophy and intestinal metaplasia cases.

### Gland segmentation

**Fig 2:**
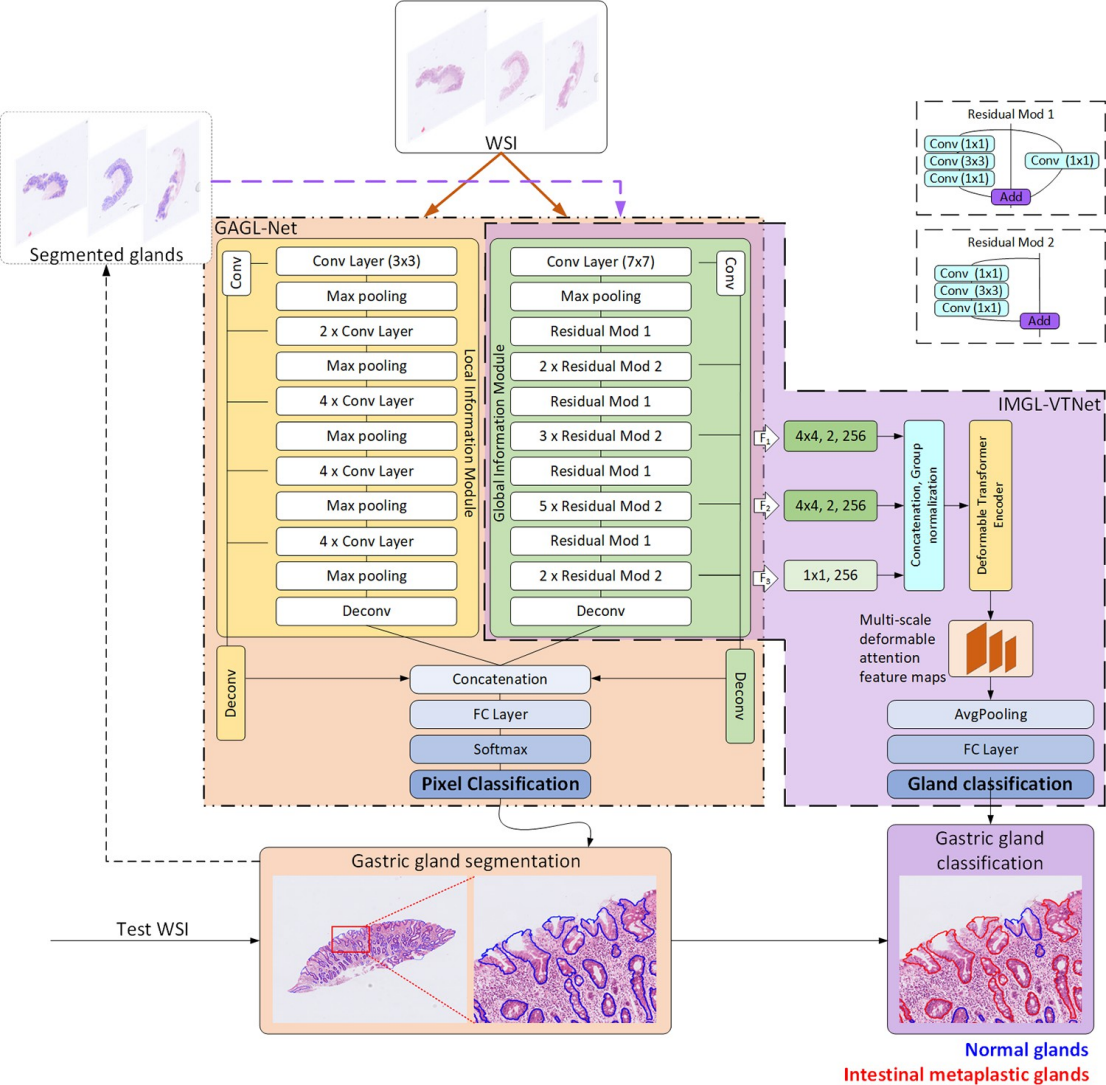
The proposed GAGL-VTNet model for gastric gland segmentation and classification.

The accurate individual identification of glands that allows the extraction of meaningful global and local features associated with gland morphology and structure remains a big challenge mainly due to the inter-observer variability. Thus, in the proposed workflow, which aims to address the aforementioned challenge, we extract different receptive field features and multi-level contextual features through a two-branch model. More specifically the segmentation part (Fig 2: GAGL-Net) of the proposed GAGL-VTNet model comprises a local module inspired by the DCAN [22] and a global module inspired by the ResNet-50 [23]. The parallel use of two modules enables the exploitation of both multi-scale local and abundant global information. In the global module the input image patches with size of 480×480×3 pass through a 7×7 conventional convolution layer while in the local module they pass through a 3×3 convolution layer. Each module includes a downsampling path and an upsampling path extracting different receptive field features. Thus, further exploitation of contextual information and finer details is incorporated. More precisely, a set of low-level features from the bottom layers is extracted that contributes to the multi-size gland segmentation. In addition, the utilized higher level features increase the overall detection accuracy of degenerated and elongated glands. In order to extract higher-level semantic information preserving the resolution, the stride and dilation of the last stage of the global module are set equal to 1 and 2 respectively. Then, the corresponding feature maps are upsampled combining convolutional and deconvolutional layers and they are concatenated to achieve pixel-level semantic segmentation. In the proposed model, the convolutional layers are followed by the batch normalization and ReLU activated function. Furthermore, a transfer learning technique is utilized initializing the layers in the downsampling path of the local and global modules with the pre-trained model parameters of VGG-19 and ResNet-50 respectively. Then, the GAGL-VTNet model is fine-tuned with training data prepared for this work.

For the training of the model, three-class labeled images are used. These represent the following categories: background, gland lumen, and gland edge. Additionally, a modified loss function is defined using a weighting factor to balance the classes.

$$Loss=L2-\sum_{p=1}^{N}w_{p}r_{p}log(t_{p})$$ (1)

where *w*_*p*_, *r*_*p*_ and *t*_*p*_ denote the weighting factors, the reference values and the predicted values at pixel *p* respectively, and *N* is the total number of pixels. L2 denotes the regularization term. Stochastic Gradient Descent (SGD) is used to optimize the loss function. The initial learn rate is defined as 0.005, the weight decay as 0.01 and the momentum as 0.9. For the testing an overlap-tile strategy for gland segmentation of WSI is used. Finally, post-processing steps, including filling holes and removing small areas, are applied aiming to improve the final segmentation output.

### Gland classification

**Fig 3:**
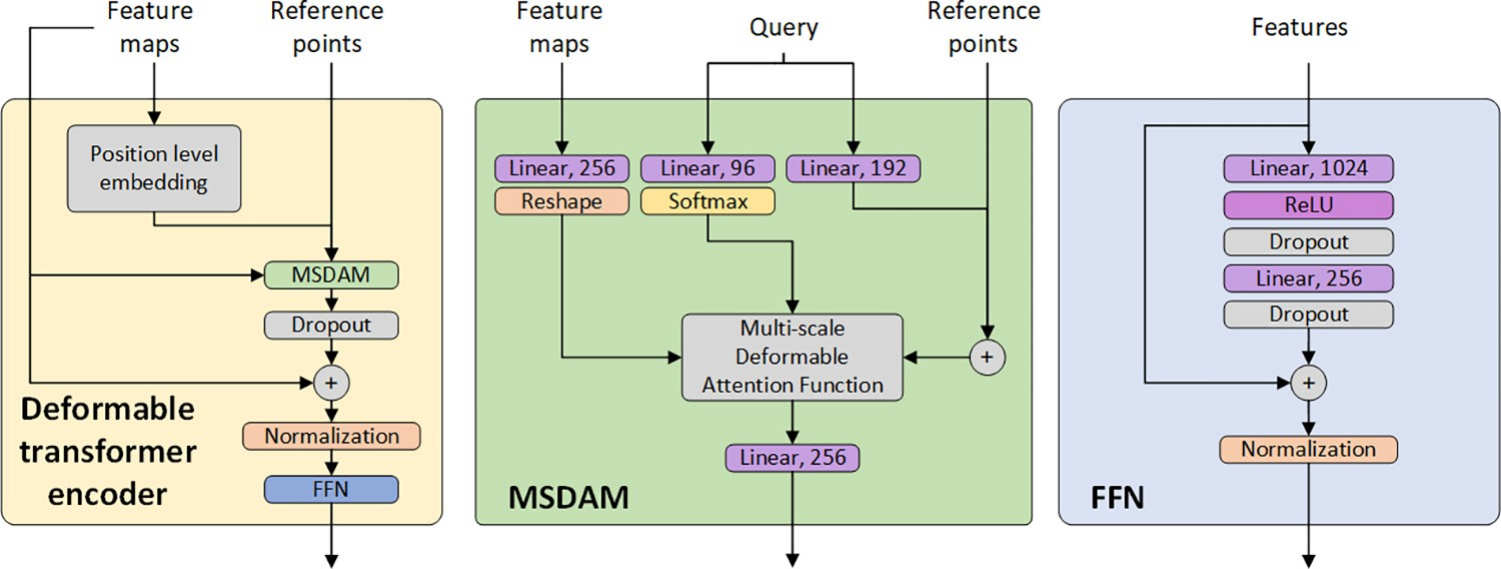
Deformable transformer encoder consisting of a Multi-scale Deformable Attention Module (MSDAM) and a Feed-Forward Network (FFN).

Following the application of the GAGL-VTNet, the masks that include the segmented glands are fed to the classification part (Fig 2: IMGL-VTNet) of the model for the discrimination of the glands into normal and IM glands. The classification model consists of the aforementioned ResNet-50 as the backbone and an adaptive feature extractor based on the deformable vision transformer. Feature maps *F*_1_ and *F*_2_ provided by the backbone are upsampled by the deconvolution layer respectively, while *F*_3_ is further embedded with a convolutional layer. Obtained multi-scale feature maps are followed by concatenation and group normalization. Then, they are sent to the multi-scale deformable vision transformer encoder (Fig 3) to adaptively capture the representative features across multi-scales and the whole feature map.

The encoder mainly consists of the Multi-Scale Deformable Attention Module (MSDAM) and a Feed-Forward Network (FFN). It takes the multi-scale feature maps and reference points as inputs. First, they are fed into the MSDAM, together with the query features which are the sum of position and scale information and the input feature maps. Then, the output of the MSDAM is added back to the input feature maps, followed by the FFN. Finally, the multi-scale enhanced features are sent to an average pooling layer followed by a fully connected layer for the classification of gastric glands into normal and IM.

To further enhance the performance of the gland classification, the following loss function *FL*_*i*_ for the *i*_*th*_ image is applied:

$${FL}_{i}=w_{focal}\cdot Loss$$ (2)

$$w_{focal}=\left\{ \begin{array}{cc} {\left(1-s\right)}^{\gamma } & p=1\\ s^{\gamma } & p=0 \end{array}\right.$$ (3)

Loss=plog(s)+(1−p)log(1−s) (4)

where binary cross-entropy and focal loss are used, *p* is the binary ground truth representing the normal or IM glands, *s* is the predicted score and *γ* = 2 is the predesigned hyperparameter in focal loss. The resulted focal loss focuses on a set of hard examples improving the precision for these cases [28]. Both in training and inference stages, the input images of the GAGL-VTNet classification part are resized and padded to (224, 224). The Adam optimizer and mean teacher method [29] are applied in training.

### Mucosa segmentation

For the analysis of the atrophy of the glands in the gastric mucosa an approach for mucosa segmentation is proposed. For the identification of the gastric mucosa which contains the glands and the gastric pits, tissue segmentation is performed. More precisely, an unsupervised energy minimization technique that is based on graph cuts is used for the training of a U-Net model. Initial labels are namely assigned to a number of pixels that are used for the annotation of the training dataset, based on a k-means clustering approach. In this labelling problem, each WSI image is represented as graph *G* = 〈*V*, *E*〉, where *V* is the set of all nodes that correspond to pixels and *E* is the set of all edges connecting adjacent nodes [30]. The labelling problem is to assign a unique label *x*_*p*_ for each node *V*, so as to minimize the following energy:

$$E=\sum_{p\in V}C_{p}(x_{p})+\sum_{(p,q)\in E}S_{p,q}(x_{p},x_{q})$$ (5)

where *C*_*p*_ is the color consistency cost which depends on the label *x*_*p*_. *S*_*p*,*q*_ is the smoothing cost between two neighboring pixels (*p*, *q*) and it depends on the labels (*x*_*p*_, *x*_*q*_). The cost of the cut which partitions the graph into two disjoined subsets, is defined to be the sum of weights of the edges crossing the cut, whereas the minimum cut problem is to find the cut with the minimum cost, that minimizes the energy either globally or locally. The algorithm results to the labelling that minimizes the energy of Eq (5) leading to the segmentation of tissue regions and background including the artefacts. Then, based on the labelling outputs, patches are created and used for the training of U-net architecture with a depth of 3. The number of feature channels is set equal to 64–128–256–512. Furthermore, patches with a size of 296×296×3 and batch size equal to 40 are used for the training. For the segmentation, overlapping patches are extracted from a WSI and fed forward into the U-net model. Finally, morphological operations for the removal of small artefacts are applied to produce the final output tissue segmentation masks.

For the mucosa segmentation morphological dilation is applied to the detected glands followed by erosion in order to both merge the glands and to keep the external boundaries of these consistent. Then, for the estimation of mucosa the output of the above transformation is combined with the tissue mask as follows:

$$M=G_{t}\cap T$$ (6)

where *G*_*t*_ is the transformed glands’ mask and the T is the tissue mask.

### Biopsies’ analysis

Following the gastric gland and mucosa segmentation, three features are extracted towards the aim of discriminating between the normal, GA and IM cases. More specifically, aiming to model the Sydney protocol and knowledge of histopathologists, the following features are extracted: i) average area that glands cover, ii) the ratio of number of glands to gastric mucosa per WSI iii) the ratio of area of glands to gastric mucosa per WSI. Subsequently, statistical analysis is performed for the identification of significant differences between normal, GA and IM cases.

### Dataset’s description

To evaluate the efficiency of the proposed methodology, a well-known dataset containing H&E-stained colorectal cancer tissue images was used. More specifically, we used the Gland Segmentation (GlaS) challenge dataset used as part of MICCAI 2015 [21]. This dataset was acquired by a team of pathologists at the University Hospitals Coventry and Warwickshire in United Kingdom. It contains 165 histological images that were extracted from 16 H&E-stained WSI. The dataset is split into the training set including 85 images (37 benign and 48 malignant), and the testing sets consisting of part A and part B which include 60 (33 benign and 27 malignant) and 20 images (4 benign and 16 malignant) respectively.

Furthermore, for the validation of the proposed workflow to gastric glands we created a dataset consisting of 85 WSI, collected from 20 patients. Gastric tissues were collected at University College London Hospital NHS trust, with ethical approval (research ethics committee (REC) reference: 15/YH/0311, & 19/LO/0089), with informed consent taken for prospective tissue collection. Samples were collected prospectively from patients undergoing gastrectomy for cancer, or sleeve gastrectomy for weight loss, with archival tissue used from endoscopic surveillance biopsies. Tissue underwent routine Hematoxylin & Eosin (H&E) staining. More specifically, the dataset includes 14 normal, 26 GA and 45 IM images. For the training of the GAGL-VTNet model we used 10 annotated WSI while for the testing we used 12 annotated WSI. Furthermore, the latter were also used for the validation of the proposed mucosa segmentation approach. For the validation of the gland classification model we used a part of the above dataset named IMGL (Intestinal Metaplasia Gastric gLands) consisting of 500 normal and 500 IM gastric glands. More specifically, we used five-fold cross validation selecting 800 gland images for the training and 200 images for the testing. For the enrichment of the training data for the tasks of gastric gland segmentation and classification an augmentation method was utilized to further increase the variability of the training dataset and to avoid overfitting of the network. In particular, we included translation, rotation and flipping transformations.

## Results and discussion

In this section, we present a detailed evaluation analysis of the proposed gastric gland and mucosa segmentation as well as gland classification. The goal of this experimental evaluation is five-fold. Initially, we compare the efficiency of GAGL-VTNet for segmentation of glands, using the publicly available colon dataset. Secondly, we use the gastric dataset developed in this study in order to validate the proposed model for the identification and classification of gastric glands on normal, GA and IM cases. In addition, the efficiency of gastric mucosa segmentation is validated. Furthermore, the proposed gland classification approach for the identification of the intestinal metaplastic is verified. Finally, the proposed workflow to the WSI gastric dataset is applied in order to analyze and determine whether significant associations could be found between the glandular morphological features of normal, GA and IM cases and whether intestinal metaplastic cases can be identified.

For the evaluation of the proposed workflow, three metrics were employed, namely F1 score, object dice and object Hausdorff [21]. The F1 score is defined as:

F1=2∙Precision∙Recall/(Precision+Recall) (7)

$$Precision=N_{TP}/(N_{TP}+N_{FP})$$ (8)

$$Recall=N_{TP}/(N_{TP}+N_{FN})$$ (9)

where *N*_*TP*_ is the number of true positive, *N*_*FP*_ is the number of false positives and *N*_*FN*_ is the number of false negatives. The F1 score corresponds to detection accuracy. The object Dice is defined as follows:

$$D_{Object}(G,S)=\frac{1}{2}[\sum_{i=1}^{n_{s}}(|S_{i}|/\sum_{p=1}^{n_{s}}|S_{p}|)D(G_{iMax},S_{i})+\sum_{j=1}^{n_{G}}(|G_{j}|/\sum_{q=1}^{n_{G}}\left|G_{q}\right|)D(G_{j},S_{jMax})]$$ (10)

where *D* is the Dice index of *G* and *S* and it is equal to D(G,S)=2(|G∩S)|)/(|G|∪|S|). *G* is the ground truth image and *S* is the segmented image. The object Dice corresponds to segmentation performance. The object Hausdorff is defined as:

$$H_{Object}(G,S)=\frac{1}{2}[\sum_{i=1}^{n_{s}}(|S_{i}|/\sum_{p=1}^{n_{s}}|S_{p}|)Η(G_{iMax},S_{i})+\sum_{j=1}^{n_{G}}(|G_{j}|/\sum_{q=1}^{n_{G}}\left|G_{q}\right|)Η(G_{j},S_{jMax})]$$ (11)

where *H* is the Hausdorff distance of *G* and *S* and it is equal to $H(G,S)=Max({sup}_{x\in G}{inf}_{y\in S}\|x-y\|,{sup}_{y\in S}{inf}_{x\in G}\|x-y\|)$. *Sup* represents the supremum and *inf* the infimum. The object Hausdorff corresponds to shape similarity. Higher score values of F1 and object Dice as well as lower scores of object Hausdorff indicate better performance.

### Comparison of gland segmentation state-of-the-art methods in colorectal tissue images

In this section, we aim to present a gland segmentation comparison of the proposed methodology using a publicly available dataset of colorectal cancer tissue images. More specifically, we use the Gland Segmentation (GlaS) challenge dataset used as part of MICCAI 2015 [21] and we compare the proposed methodology against a number of different gland segmentation approaches.

More specifically, in Table 1, we present the evaluation results of the GAGL-VTNet model in comparison to thirteen state-of-the-art methods. This analysis reveals that GAGL-VTNet is amongst the top performing methods. More precisely, the proposed model towards gland segmentation achieves F1 score rates of 0.918 and 0.855 for the part A and part B test sets respectively. The achieved F1 score for part A is the second-best rate while for part B the proposed model achieves the top performance. Similarly, the achieved object Dice rates are 0.915 and 0.854 for part A and part B respectively. These rates correspond to the top performance and to the second-best score against the compared methods respectively. Moreover, the proposed model achieves object Hausdorff scores of 41.48 and 98.96 corresponding to the second and third-best performances for part A and part B test sets respectively.

**Table 1:** Performance comparison to other methods.

*Method*	*F1 Score*		*Object Dice*		*Object Hausdorff*	
	*Part A*	*Part B*	*Part A*	*Part B*	*Part A*	*Part B*
*CVML [21]*	0.652	0.541	0.644	0.654	155.43	176.24
*LIB [21]*	0.777	0.306	0.781	0.617	112.71	190.45
*FCN-8 [31]*	0.783	0.692	0.795	0.767	105.04	147.28
*SegNet [32]*	0.858	0.753	0.864	0.807	62.62	118.51
*DeepLab v3 [33]*	0.862	0.764	0.859	0.804	65.72	124.97
*Freidburg2 [21]*	0.87	0.695	0.876	0.786	57.09	148.47
*Manivannan et al. [34]*	0.892	0.801	0.887	0.853	51.175	**86.987**
*Xu et al. [23]*	0.893	0.843	0.908	0.833	44.13	116.82
*ExB3 [21]*	0.896	0.719	0.886	0.765	57.36	159.87
*CUMedVision2 [22]*	0.912	0.716	0.897	0.781	45.42	160.35
*MILD-Net [24]*	0.914	0.844	0.913	0.836	41.54	105.89
*TCC-MSFCN [26]*	0.914	0.850	0.913	**0.858**	**39.84**	93.24
*Yan et al. [25]*	**0.924**	0.844	0.902	0.840	49.881	106.075
***GAGL-VTNet***	0.918	**0.855**	**0.915**	0.854	41.48	98.96

**Fig 4:**
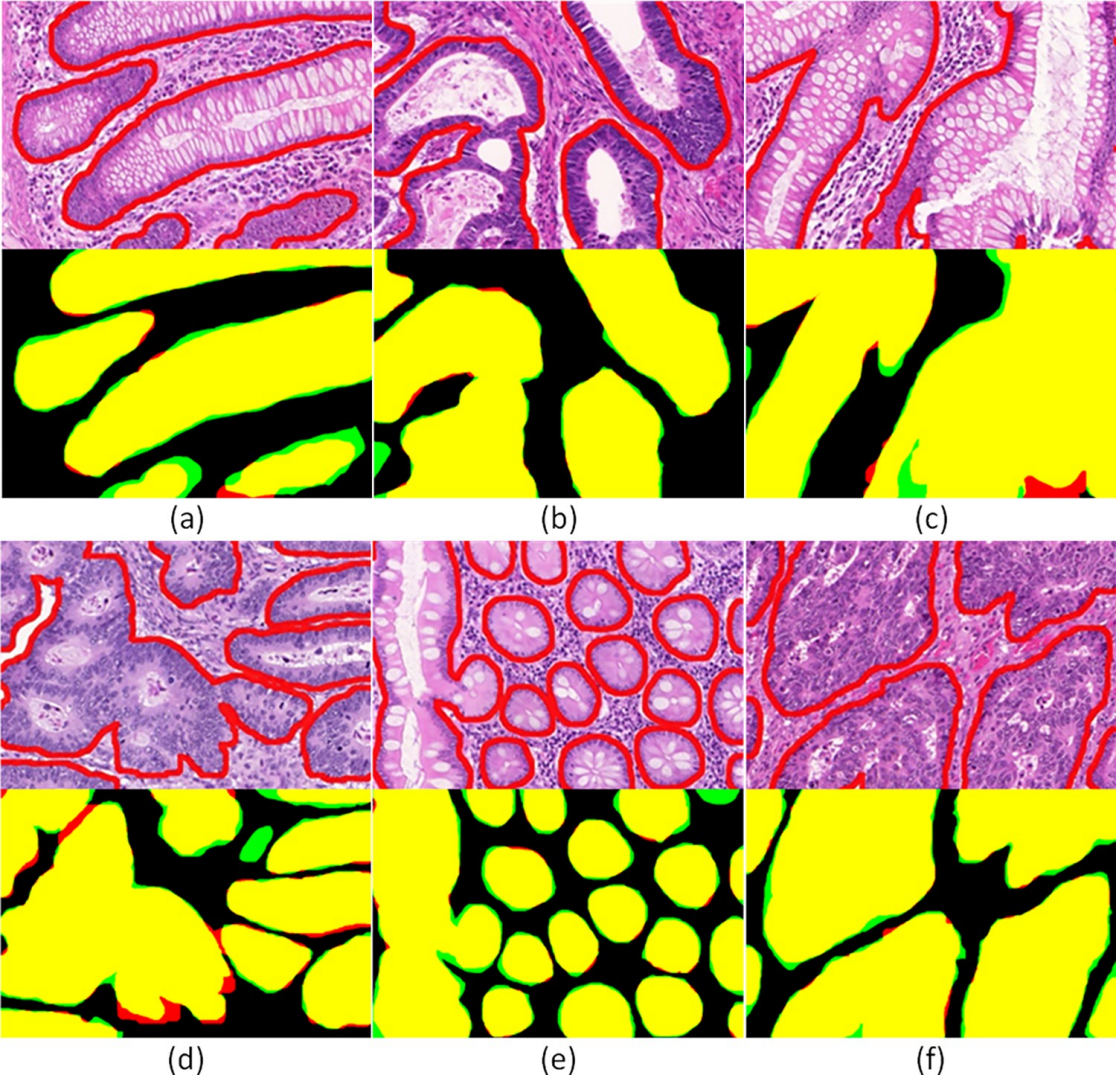
Gland segmentation results of GAGL-VTNet model on the GlaS challenge dataset in comparison with ground truth: Yellow color (true positive), red color (false positive), green color (false negative).

It is worth mentioning that the top performing models combine similar techniques and properties in order to achieve accurate gland segmentation. These include the extraction of different receptive field features, the use of weighted loss functions and the simultaneous gland segmentation and boundary detection. From Table 1, we can infer that the proposed model, combining a global and a local branch and simultaneously extracting different receptive field features, offers an improved F1 score for malignant cases as well as a higher score in segmentation performance of benign cases. In contrast, although some models [26, 34] achieve better shape similarity (object Hausdorff score), they achieve lower detection (F1 score) and segmentation (object Dice score) rates that would lead to inaccurate results regarding the estimation of the number and area of glands that are used in this workflow for the analysis of GA and IM cases. Fig 4 illustrates qualitative results of the proposed model on the GlaS challenge dataset. It shows that in the most cases the GAGL-VTNet model accurately identifies both benign and malignant glands. However, there is a limited number of cases where the lack of lumen (Fig 4D) or the existence of glands in the border of the images (Fig 4E) cause false negative results.

### Gland and mucosa segmentation in gastric tissue images

**Fig 5:**
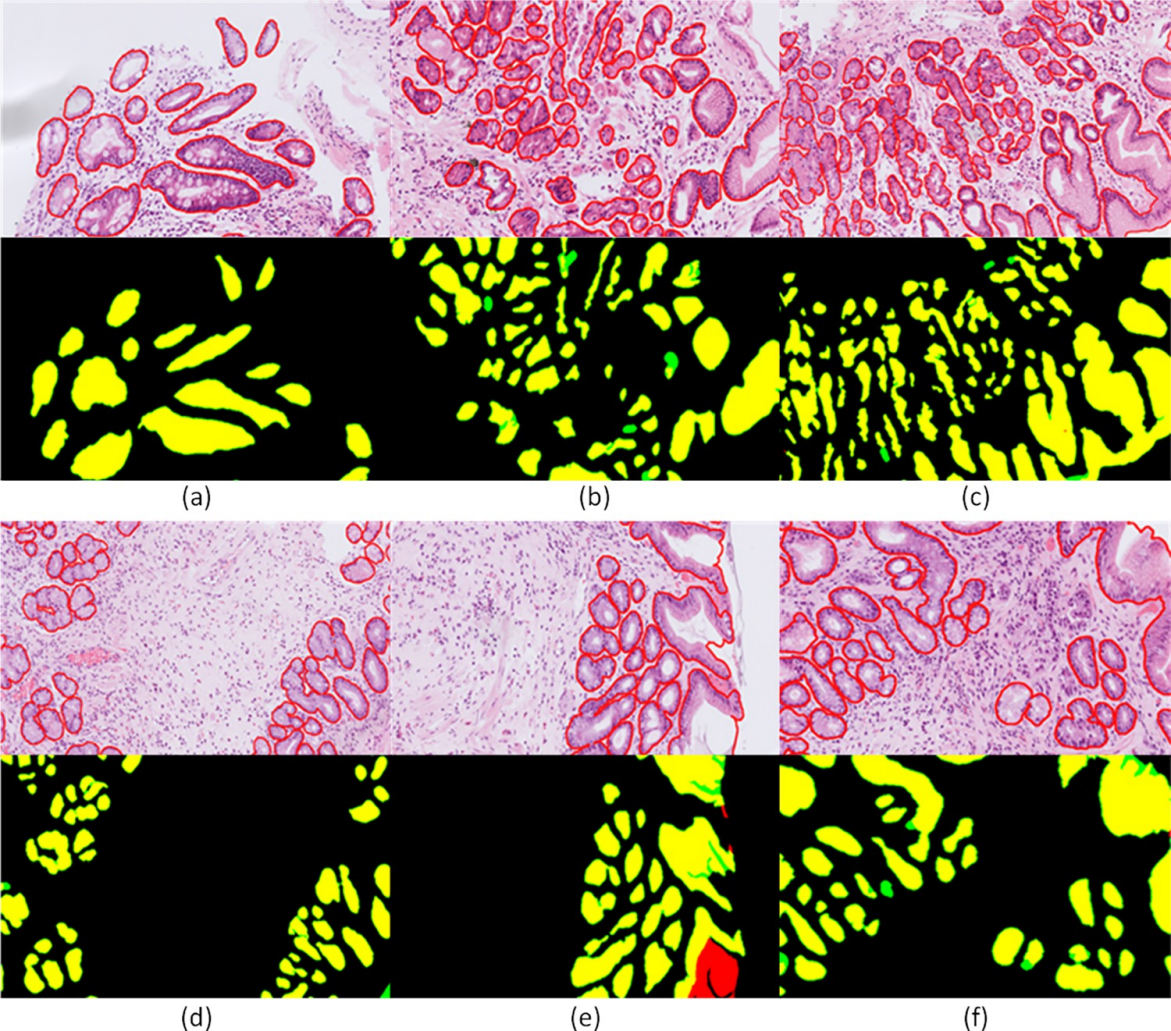
Gland segmentation results of GAGL-VTNet model on GAGL dataset in comparison with ground truth: Yellow color (true positive), red color (false positive), green color (false negative).

Subsequently, in order to confirm that the performance of the proposed methodology remains robust in gastric tissues, we carried out a validation analysis using the GAGL dataset. More specifically, we used 12 annotated WSI and the GAGL-VTNet model in order to perform segmentation of the gastric glands and the gastric pits. The results shows that the proposed gland segmentation approach achieves F1 score equal to 0.914 and object Dice score equal to 0.908. Moreover, the proposed model achieves object Hausdorff score equal to 44.12. Similarly to the GlaS dataset, results in the GAGL dataset (Fig 5) show the great potential of the proposed model that is capable of identifying glands with high shape and size diversity. However, there is a limited number of small glands and gastric pits that are not accurately detected due to either the small size of glands or image artefacts.

**Fig 6:**
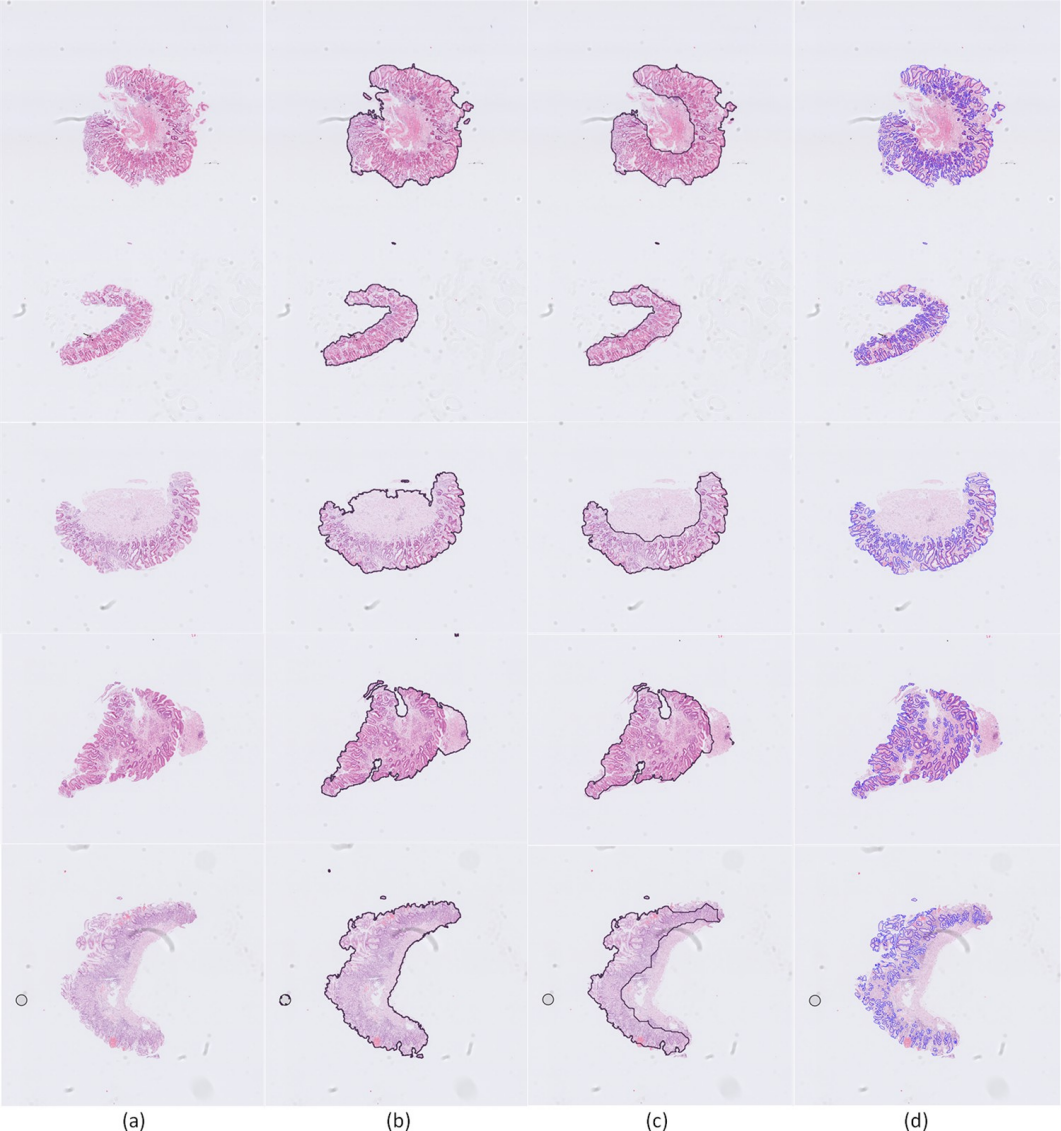
Qualitative results of the segmentation of the gastric tissue, gastric mucosa and gastric glands: a) input 10x H&E-stained image, b) gastric tissue segmentation, c) gastric mucosa identification, d) gastric glands detection.

The mucosa segmentation approach achieves F1 score and dice score equal to 1 and 0.967 respectively. Fig 6 demonstrates that the proposed methodology accurately identifies the gastric mucosa in all the studied cases. More specifically, Fig 6A shows the input H&E images that are used for the analysis. The output of the U-Net model for tissue segmentation is shown in Fig 6B while the identification of the gastric mucosa is shown in Fig 6C. The detected gastric glands are shown in Fig 6D. Furthermore, results in Fig 6 show that the proposed methodology provides accurate segmentation even in the presence of scanning and image artefacts.

### Gland classification in gastric tissue images

In this section, we present a comparison of the proposed gland classification model against a number of classification approaches. More precisely, in Table 2, we present the evaluation results of the GAGL-VTNet classification model in comparison to seven classification models. For the comparison, we used the IMGL dataset and we considered the most widely used models that have been applied to various tasks.

**Table 2:** Comparison of gland classification using different models.

*Method*	*Precision*	*Recall*	*F1 score*
*ResNet-18*	0.92±0.04	0.84±0.03	0.88±0.03
*ResNet-50*	0.91±0.03	0.86±0.03	0.89±0.03
*ResNet-101*	0.91±0.03	0.82±0.03	0.86±0.03
*VGG-19*	0.89±0.03	0.89±0.02	0.88±0.02
*Inception-V3*	0.91±0.04	0.81±0.03	0.86±0.04
*Xception*	0.82±0.05	0.78±0.04	0.79±0.04
*BotNet-50*	0.92±0.03	0.90±0.02	0.91±0.02
***GAGL-VTNet***	**0.95±0.03**	**0.94±0.02**	**0.94±0.03**

**Fig 7:**
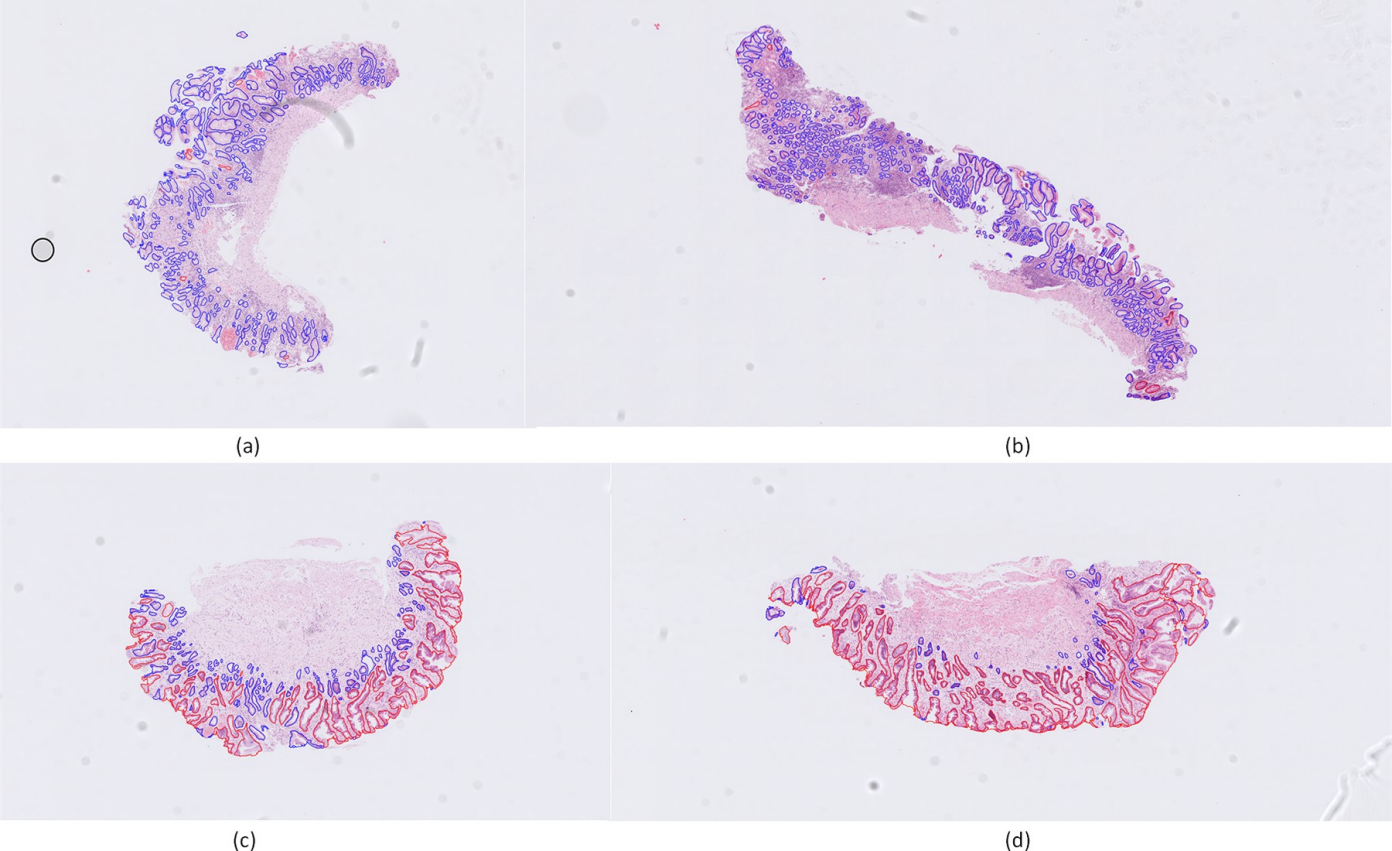
Gland classification using the GAGL-VTNet model on four WSI: a-b) normal cases, c-d) IM cases. Blue color denotes the glands that have been detected as normal and red color denotes the glands that have been detected as IM glands.

The results (Fig 7) show that the proposed gland classification approach achieves precision equal to 0.95 and recall equal to 0.94. Moreover, the proposed model achieves F1 score equal to 0.94. The proposed model achieves an F1 score improvement of 0.06 to the widely used VGG-19 and 0.05 compared to the ResNet-50. Furthermore, the application of the BotNet-50 that combines ResNet-50 with a Multi Head Self-Attention (MHSA) layer, improves the F1 score by 0.02, compared to ResNet-50. Thus, the proposed model improves the F1 score by 0.03 compared to the BotNet-50.

### Gastric biopsies analysis

**Fig 8:**
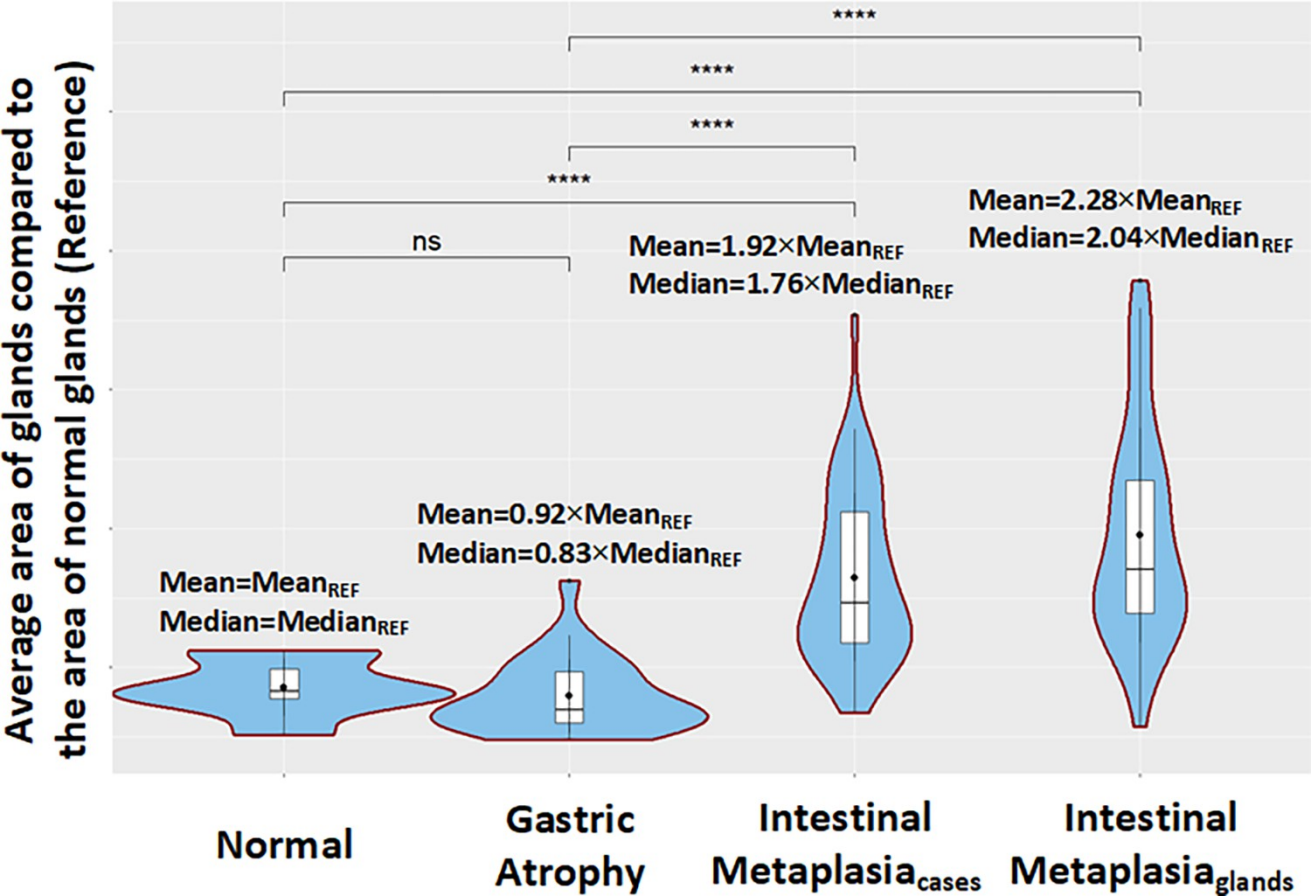
Box plots comparing the average area that glands cover per WSI between normal, Gastric Atrophy (GA) and Intestinal Metaplasia (IM) cases included in GAGL dataset. For the comparison the average area of normal glands is used as reference. For IM cases, average area of glands per IM case and average area of glands classified as IM is estimated.

In this work, features of the segmented and classified glands as well as segmented mucosa are used for the analysis of gastric biopsies. Initially, we estimate the average area that glands cover per WSI and using as reference the average area of normal glands we compare the normal, GA and IM cases (Fig 8). It is worth mentioning that for IM cases, a twofold analysis is performed. Firstly, we estimate the average area of glands per IM case and then we calculate the average area of glands classified as IM for these cases. More specifically, the average area of total number of glands in GA cases is equal to 0.92 times of the reference average. The average area of glands in IM cases is 1.92 times of the reference area while the average area of IM glands in IM cases is 2.28 times of the average area of normal glands. Moreover, we have carried out tests and significant differences between normal and IM cases as well as between GA and IM have been identified. Furthermore, in accordance with the Sydney system, statistically non-significant differences have been identified between normal and GA cases. These results validate the remarks of the visual analogue scale introduced in that system and quantify the differences between glands’ areas of GA, IM and normal cases.

**Fig 9:**
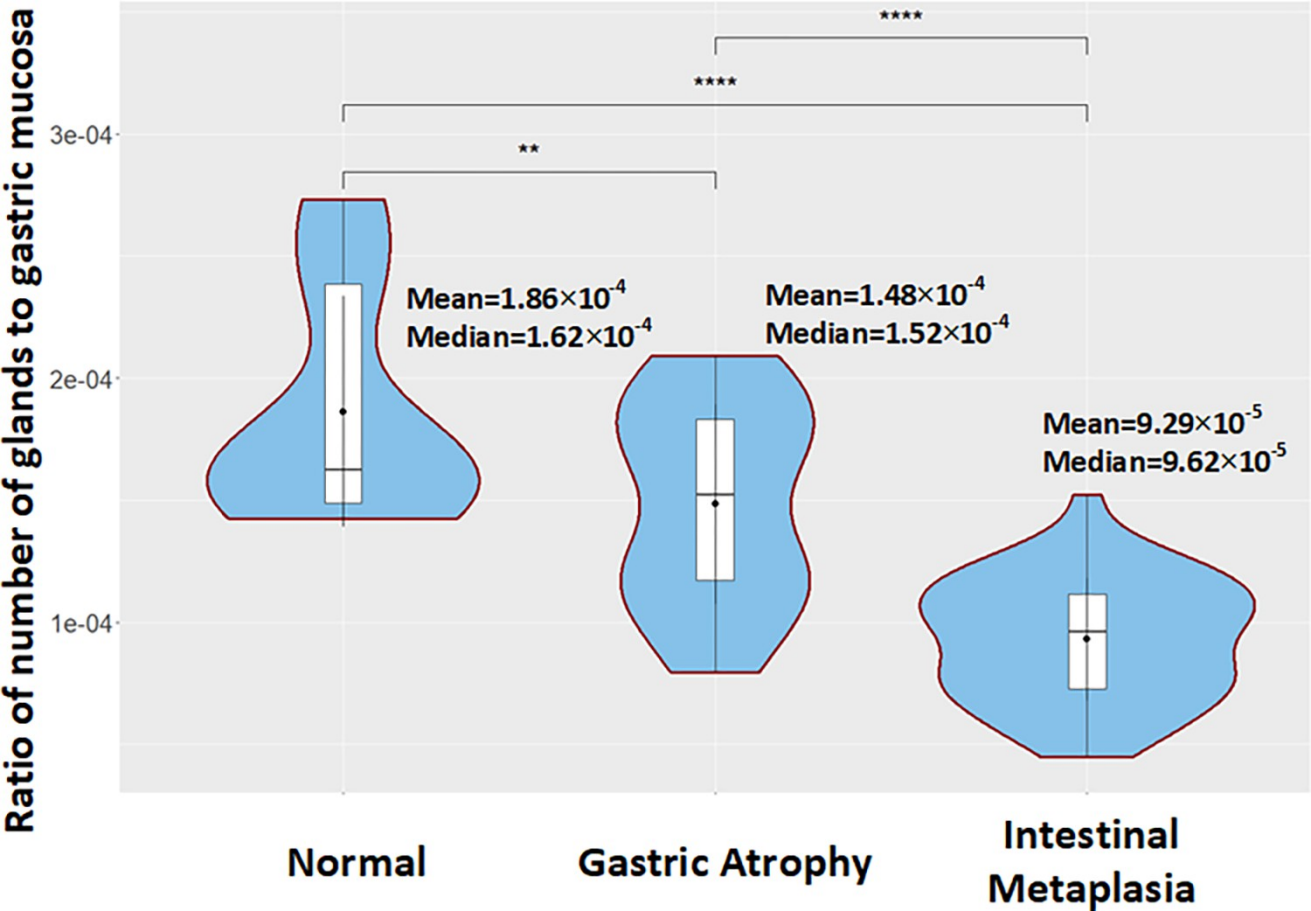
Box plots comparing the ratio of number of glands to gastric mucosa between normal, Gastric Atrophy (GA) and Intestinal Metaplasia (IM) cases included in the GAGL dataset.

In additional analyses, we estimate the ratio of the number of glands to gastric mucosa (Fig 9). Thus, taking into account the number of identified glands and the area of segmented mucosa we estimate the aforementioned ratio for normal cases to be equal to 1.86×10^−4^. For GA cases the ratio is estimated to be equal to 1.48×10^−4^ and for the IM cases equal to 9.29×10^−5^. It is worth mentioning that the statistical analysis of biopsy specimens reflects the expected loss of glands in the gastric mucosa. Furthermore, the results validate the fact that IM cases are usually associated with extensive atrophy carrying an increased risk of malignancy [6].

**Fig 10:**
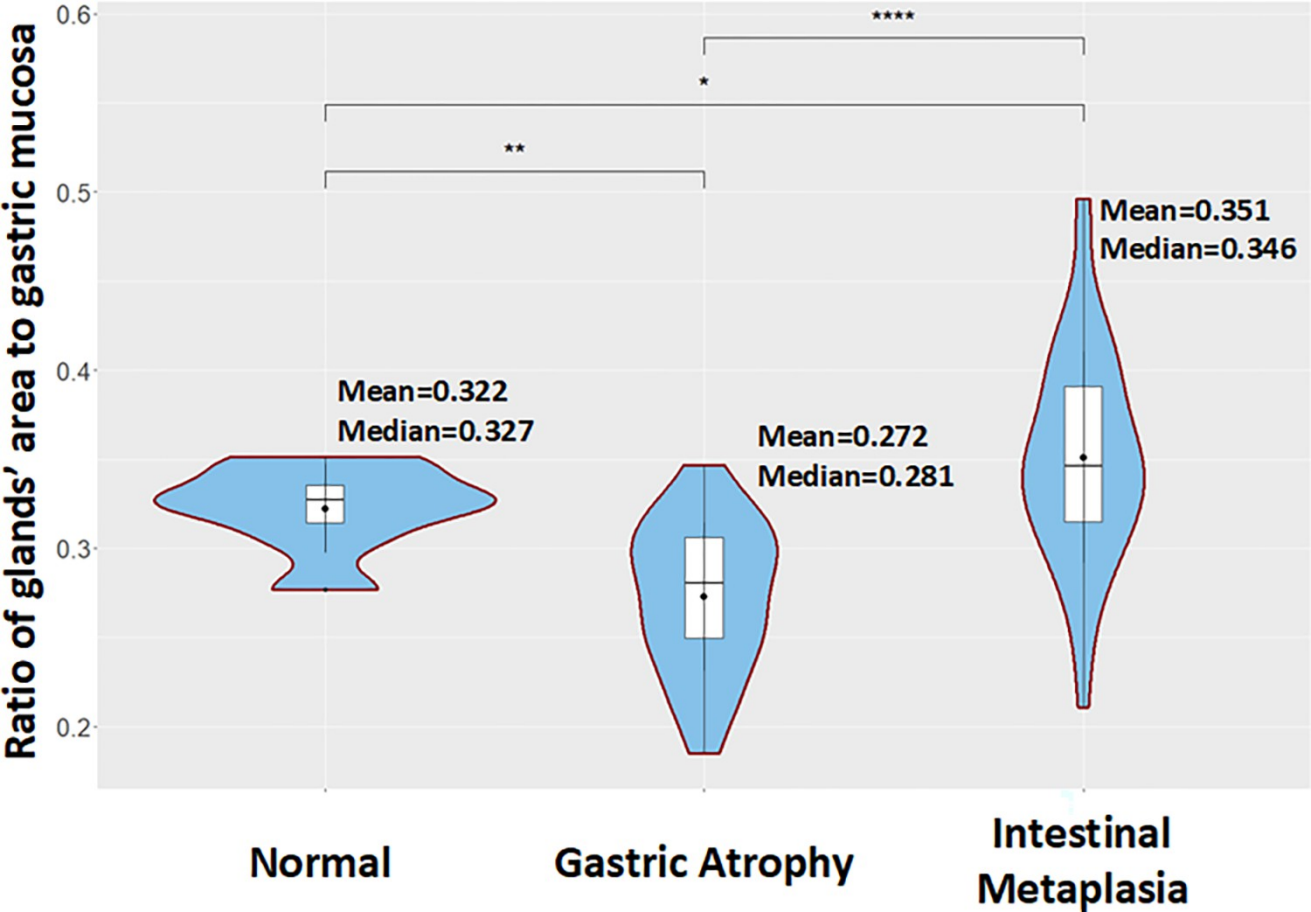
Box plots comparing the ratio of the area covered by glands to gastric mucosa between normal, Gastric Atrophy (GA) and Intestinal Metaplasia (IM) cases included in the GAGL dataset.

Finally, we estimate the ratio of the area of glands to gastric mucosa per WSI (Fig 10). More specifically, we calculate the summary of the area of glands for each WSI and we divide it by the gastric mucosa area. The average ratio for the normal cases is found to be equal to 0.322, for the atrophic cases the average ratio is estimated to be equal to 0.272 while for the IM cases it is determined to be equal to 0.351. It is worth mentioning that significant differences are identified between all the different cases. Furthermore, the lowest ratio value is observed in atrophic cases while the larger glands of IM cases led to higher average ratio scores.

It is worth mentioning that the aforementioned analysis not only provides a fast and reliable method to assist the analysis and diagnosis of GA and IM but also could possibly lead to its widespread adoption in routine histopathological practice. Furthermore, these results contribute to the translation of the visual recognition of atrophic and IM by histopathologists through the Sydney system into a reproducible set of mathematical values regarding the average area of glands, the ratio of number of glands to gastric mucosa and the ratio of summarized area of glands to gastric mucosa.

## Conclusions

Multiple risk factors and a multistep process have been associated with gastric carcinogenesis. Among these factors, gastric atrophy (GA), and gastric intestinal metaplasia (IM) of the mucosa have been recognized as high-risk precancerous lesions for dysplasia and gastric cancer. However, as the manual assessment of biopsies by histopathologists based on the Sydney System is a laborious and time-consuming task, the early and accurate detection of GA and IM necessitates the adoption of artificial intelligence methods. Thus, in this paper we propose a methodology for the automated analysis of gastric tissue biopsies including glands and mucosa segmentation as well as glands’ classification. The proposed models for gastric gland segmentation and the mucosa segmentation method achieve F1 score equal to 0.914 and 1 respectively. Similarly, they achieve object Dice score equal to 0.908 and 0.967 for gland and mucosa segmentation respectively. Furthermore, the proposed classification model achieves F1 score equal to 0.94.

The results suggest that the proposed workflow not only obtains good segmentation and classification performance on the GAGL dataset but also shows an excellent generalization ability on the widely used GLAS dataset. The analysis of tissue biopsies reflects the expected results based on the Sydney scoring system and through this a set of mathematical values for the standardisation of studied precancerous lesions is provided. The presented workflow and results can be used in routine pathology in order to serve as a relevant diagnostic parameter as well as in future studies. However, limitations of this study include the lack of analysis regarding the biopsy sites and more detailed analysis with regards to the histological grading. Thus, a future step would include the use of the proposed workflow for the analysis of WSI that have been received from greater and lesser curvature of the antrum and corpus mucosa. Finally, future studies are needed to prove that this methodology will be validated in gastric tissue biopsies from other centers in order for the proposed framework to be adopted on a widespread basis in routine histopathological practice.
